# Implication of Dietary Iron-Chelating Bioactive Compounds in Molecular Mechanisms of Oxidative Stress-Induced Cell Ageing

**DOI:** 10.3390/antiox10030491

**Published:** 2021-03-21

**Authors:** Alexandra Barbouti, Nefeli Lagopati, Dimitris Veroutis, Vlasios Goulas, Konstantinos Evangelou, Panagiotis Kanavaros, Vassilis G. Gorgoulis, Dimitrios Galaris

**Affiliations:** 1 Department of Anatomy-Histology-Embryology, Faculty of Medicine, School of Health Sciences, University of Ioannina, 45110 Ioannina, Greece; pkanavar@uoi.gr; 2 Laboratory of Histology-Embryology, Molecular Carcinogenesis Group, Faculty of Medicine, School of Health Science, National and Kapodistrian University of Athens, 75, Mikras Asias Str., Goudi, 11527 Athens, Greece; nlagopati@med.uoa.gr (N.L.); dimitrisveroutis1@gmail.com (D.V.); cnevagel@med.uoa.gr (K.E.); vgorg@med.uoa.gr (V.G.G.); 3 Department of Agricultural Sciences, Biotechnology and Food Science, Cyprus University of Technology, 3036 Lemesos, Cyprus; vlasios.goulas@cut.ac.cy; 4 Biomedical Research Foundation Academy of Athens, 11527 Athens, Greece; 5 Faculty of Biology, Medicine and Health Manchester Cancer Research Centre, Manchester Academic Health Sciences Centre, University of Manchester, Manchester M13 9PL, UK; 6 Center for New Biotechnologies and Precision Medicine, Medical School, National and Kapodistrian University of Athens, 11527 Athens, Greece; 7 Laboratory of Biological Chemistry, Faculty of Medicine, School of Health Sciences, University of Ioannina, 45110 Ioannina, Greece; dgalaris@uoi.gr

**Keywords:** ageing mechanisms, bioactive dietary compounds, cellular senescence, free radicals, iron-chelating agents, labile iron, Mediterranean diet, oxidative stress

## Abstract

One of the prevailing perceptions regarding the ageing of cells and organisms is the intracellular gradual accumulation of oxidatively damaged macromolecules, leading to the decline of cell and organ function (free radical theory of ageing). This chemically undefined material known as “lipofuscin,” “ceroid,” or “age pigment” is mainly formed through unregulated and nonspecific oxidative modifications of cellular macromolecules that are induced by highly reactive free radicals. A necessary precondition for reactive free radical generation and lipofuscin formation is the intracellular availability of ferrous iron (Fe^2+^) (“labile iron”), catalyzing the conversion of weak oxidants such as peroxides, to extremely reactive ones like hydroxyl (HO^•^) or alcoxyl (RO^•^) radicals. If the oxidized materials remain unrepaired for extended periods of time, they can be further oxidized to generate ultimate over-oxidized products that are unable to be repaired, degraded, or exocytosed by the relevant cellular systems. Additionally, over-oxidized materials might inactivate cellular protection and repair mechanisms, thus allowing for futile cycles of increasingly rapid lipofuscin accumulation. In this review paper, we present evidence that the modulation of the labile iron pool distribution by nutritional or pharmacological means represents a hitherto unappreciated target for hampering lipofuscin accumulation and cellular ageing.

## 1. Introduction

Natural ageing represents a process in which multiple degenerative molecular mechanisms are implicated, leading to the progressive general decline of organ functions. Ageing is accompanied by phenotypic changes that are related to both genetic and epigenetic factors, ultimately causing structural disorganization, functional decline, and an increased probability of diseases and death. It is plausible to imagine that the elucidation of the underlying complex biochemical mechanisms that determine the rate of biological ageing should be of utmost clinical importance [1].

The most attractive theory for explaining the ageing process is the so called “free radical theory of ageing” proposed in 1956 by Denham Harman [2]. This theory suggested that some of the oxygen-derived reactive free radicals generated in aerobic cells can escape the surveillance of protective defense mechanisms, leading to the non-specific oxidation of all basic cell constituents (proteins, lipids, nucleotides, carbohydrates etc.).

Cells have developed sophisticated systems that can both rapidly remove oxygen-derived oxidants and detect and repair their oxidatively damaged components. However, in cases of increased and long-lasting oxidative stress conditions, the capacity of cells to repair their damaged parts can reach saturation, allowing for the further oxidation of the already oxidized components and the accumulation of over-oxidized non-repairable material inside the cells. This phenomenon causes modifications of the overall cell structure and challenges normal cell function, as is apparent in ageing and senescence [3].

The exact molecular mechanisms underlying the generation of highly reactive free radicals that are able to damage cellular constituents and promote the accumulation of unrepairable material remain poorly understood. The elucidation of these mechanisms should certainly provide useful ideas and molecular tools for intervening in the ageing process and probably preventing the development of ageing-related diseases [4].

A necessary precondition for the production of highly reactive free radicals inside cells is the availability of ferrous iron ions (Fe^2+^), which can catalyze the conversion of weak oxidants such as peroxides to extremely reactive ones like hydroxyl (HO^•^) or alcoxyl (RO^•^) radicals. This part of cellular iron represents a small percentage of the total cellular iron and is usually called “labile iron” [5,6]. Thus, the depletion or redistribution of intracellular labile iron by exogenous compounds may diminish the formation of damaging reactive radicals in the case of increased oxidative stress and prevent the oxidation and over-oxidation of cellular components. Interestingly, a plethora of iron-chelating bioactive compounds have been shown to be present in the Mediterranean type of diet [7,8,9,10]. Moreover, it has been proven that when these agents can reach the cell interior, they protect cells from damage in conditions of oxidative stress [11,12].

In the present review article, we focus our interest on describing the chemical interactions that contribute to the oxidation and over-oxidation of cellular constituents. Special attention is paid to the key role of labile iron (redox-active iron) in these processes, as well as the potential involvement of dietary natural iron-chelating bioactive compounds in controlling the level and/or the spatial distribution of intracellular labile iron.

## 2. Reactive Oxygen Species and the Concept of Oxidative Stress

### 2.1. The Oxygen Paradox

Oxygen is indispensable for life, and, except for certain anaerobes, all animals, plants, and bacteria require oxygen to grow. The main function of oxygen in aerobes is to serve as the terminal acceptor of the electrons in the final step of mitochondrial electron transport chain, which represents the pivotal process in energy-producing oxidative catabolism. However, oxygen’s chemical properties predispose it to the generation of highly reactive oxygen intermediates that can oxidize essential cell components, jeopardizing cellular and, by extension, organismal homeostasis. Hence, there is a curious paradox: oxygen is indispensable for aerobes, while, at the same time, its metabolic byproducts are unavoidable and potentially toxic. It is apparent that the production and removal of these species constantly occur within cells, keeping them at basal non-toxic levels [5]. However, under certain circumstances, this finely regulated balance can be disrupted. If the rate of their formation exceeds that of their removal, the steady state concentrations should be elevated, thus increasing the probability for the generation of potentially damaging reactive free radicals, a state known as “oxidative stress” [13,14].

In this part, we provide a brief description of the concept of “oxidative stress” based on the biochemical mechanisms of the intracellular formation and removal of reactive oxygen intermediates, with a special focus on the generation of extremely reactive free radicals that can indiscriminately oxidize all basic cell constituents.

### 2.2. Reactive Oxygen Species and Oxidative Stress

**Figure 1:**
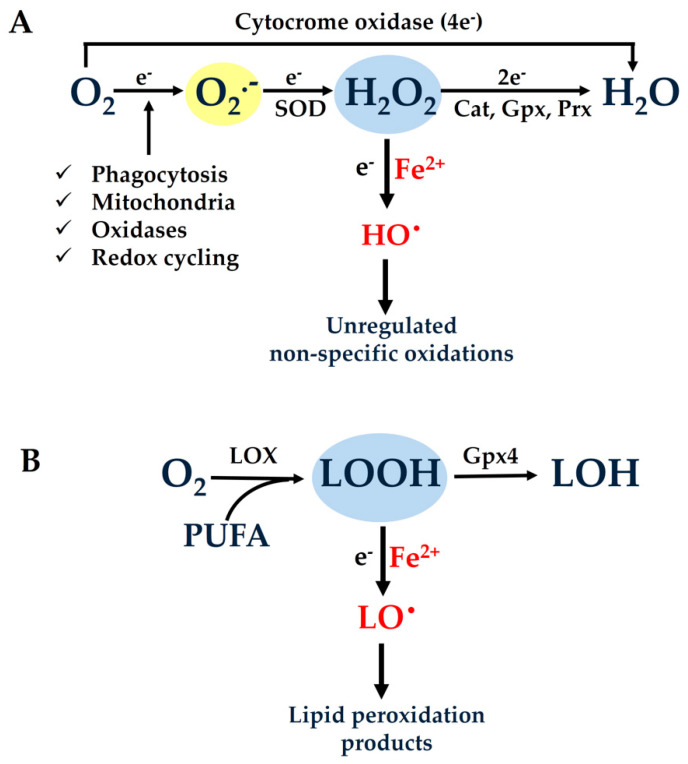
(**A**) Most of the oxygen (O_2_) consumed by aerobic organisms is reduced with four electrons to H_2_O in the last step of the mitochondrial respiratory electron transport chain by the enzyme cytochrome oxidase (complex IV). However, a small portion of O_2_ undergoes single electron reduction, thus producing superoxide anion (O_2_^•−^), which is rapidly converted to hydrogen peroxide (H_2_O_2_) by the action of the enzyme superoxide dismutase (SOD). The generated H_2_O_2_ is further reduced, either enzymatically by two electrons to H_2_O through the action of the enzymes catalase (Cat), glutathione peroxidase (Gpx), and peroxiredoxin (Prx), or non-enzymatically by one electron, thus leading to the generation of extremely reactive hydroxyl radicals (HO^•^). The latter reaction, which requires available ferrous iron (Fe^2+^), is known as the “Fenton reaction” and generates reactive intermediates that are able to indiscriminately oxidize cell components. (**B**) Lipids and fatty acids located in membranes and lipoproteins can also incorporate O_2_ and be peroxidized by the action of the enzyme “lipoxygenase” (LOX). The generated lipoperoxides (LOOHs) can be reduced to the corresponding alcohols by the specific membranous enzyme “glutathione peroxidase 4” (Gpx4). Like H_2_O_2_, LOOHs can interact with available ferrous iron (labile iron), leading to the generation of highly reactive lipid alcoxyl radicals (LO^•^s), which can further promote the peroxidation of membrane lipids.

As mentioned above, the four-electron reduction of molecular oxygen is catalyzed by the last enzyme in the respiratory electron transport chain, named cytochrome oxidase. During this reaction, the enzyme receives four electrons, one from each of four cytochrome c molecules, and transfers them to molecular oxygen (O_2_) (Figure 1A). Thus, most of the O_2_ consumed by aerobic organisms is reduced to water in the respiratory chain within the mitochondrial inner membrane without causing collateral damage.

However, a small amount of consumed O_2_, even under physiological conditions, undergoes single electron reduction to produce superoxide anions (O_2_^•−^), which are rapidly converted to hydrogen peroxide (H_2_O_2_) by superoxide dismutases (SODs) (Figure 1A). The generated H_2_O_2_ can be further reduced, either enzymatically by two electrons to H_2_O or non-enzymatically by one electron to lead to the production of extremely reactive hydroxyl radicals (HO^•^). The latter reaction requires available ferrous iron (Fe^2+^) and is known as the “Fenton reaction” [15].

Apart from H_2_O_2_, lipid hydroperoxides (LOOHs) are also normally generated through the action of the enzyme “lipoxygenase” (LOX) (Figure 1B). A specific membrane-bound “glutathione peroxidase 4” (Gpx4) is responsible for the removal of excess LOOHs [16]. Like H_2_O_2_, LOOHs can interact with Fe^2+^, thus leading to the generation of highly reactive lipid alcoxyl radicals (LO^•^s). These species can further promote chain reactions that intensify the process of lipid peroxidation and the production of aldehydes as final stable products. Interestingly, it was recently shown that the improper function of Gpx4 in combination with elevated levels of available Fe^2+^ invariably leads to a distinct type of regulated cell death named “ferroptosis” [17].

All the aforementioned intermediates of O_2_ reduction are collectively called reactive oxygen species (ROS). It has to be stressed, however, that the term ROS itself contains an inherent contradiction because it comprises both weak oxidants such as O_2_^•−^ and H_2_O_2_ and extremely reactive ones such as HO^•^ and RO^•^ [5]. In addition, the elevation of ROS in conditions of oxidative stress is not simultaneous for all these species, but the generation of reactive HO^•^ and RO^•^ depends on the presence or the absence of ferrous iron. It is obvious from the above considerations that the presence of available labile iron plays a pivotal role for the generation of highly reactive free radicals during conditions of increased rates of hydroperoxide formation (oxidative stress). Thus, controlling the concentration of available Fe^2+^ has arisen as a rational strategy for the effective protection of cells in conditions of oxidative stress [18]. Such a strategy should primarily aim to prevent the generation of HO^•^s and RO^•^s rather than scavenging them after they are formed, which seems impossible due their high-rate constants of reaction.

### 2.3. Mechanisms of ROS Generation and Removal

The partial reduction of O_2_ can be facilitated by the activation of several mechanisms in mammalian cells [14]. The most important factor from a quantitative point of view is the enzyme NADPH oxidase 2 (Nox2), which is located on the plasma membrane of professional phagocytes. When activated, Nox2 can produce excessive amounts of O_2_^•−^ and many other downstream reactive species [19] that aim to kill invading foreign microorganisms in sites of inflammation and infections. Under these conditions, professional phagocytes are attracted and activated, leading to dramatic increases of O_2_ consumption (about 100 fold), a fact usually called “respiratory” or “oxidative” burst. The produced O_2_^•−^ can trigger the initiation of several complex biochemical pathways that lead to the further formation of strong oxidants that are able to extinguish potential microbial invaders [20,21]. Apart from Nox2, several other members of the NADPH oxidase family (Nox1, Nox3-5, and DUOX1-2) can generate limited amounts of O_2_^•−^ when activated, mainly for signaling purposes [22].

Mitochondria are also a major intracellular source of reactive oxygen intermediates. Electron transfer complexes—especially complex I and complex III in the respiratory chain—may leak electrons to O_2_, which is partially reduced to O_2_^•−^ [23,24]. A variety of other oxidases prominently present in different cellular compartments are also able to produce reactive oxygen intermediates. In addition, oxygen-derived reactive byproducts can be generated from interactions with exogenous sources such as environmental pollution, drugs, ionizing, solar radiation, and nutrients (Figure 1A).

During evolution, aerobic cells developed sophisticated antioxidant defense mechanisms in order to rapidly eliminate the continuously generated weak oxygen-derived oxidants such as O_2_^•−^ and H_2_O_2_. The scavenging enzymes that metabolize these intermediates are regarded as the first line of defense in protecting cells exposed to conditions of oxidative stress [25]. Thus, O_2_^•−^ is rapidly converted to H_2_O_2_ via SODs, while H_2_O_2_ can be eliminated by enzymes such as catalases (Cats), Gpx, and peroxiredoxins (Prx) (Figure 1A).

Both O_2_^•−^ and H_2_O_2_, which represent the one- and two-electron reduction products of oxygen, respectively, are moderately reactive and can only directly interact with a limited number of cellular molecules, mainly iron–sulfur (4F–4S) cluster-containing proteins, leading to the liberation of labile iron and the modulation of the activity of the corresponding proteins [26]. On the contrary, HO^•^s and RO^•^s that are generated after interaction of H_2_O_2_ or ROOH with Fe^2+^ exhibit an extremely high reactivity. Actually, HO^•^ is regarded as one of the most reactive molecules produced in living cells, as it is able to instantly and indiscriminately oxidize whatever chemical group happens to be in the vicinity of its generation (diffusion-controlled reactivity) [5]. The necessary parameter for the generation of HO^•^s and RO^•^s is the simultaneous presence of elevated levels of H_2_O_2_ or ROOH with Fe^2+^ for an adequate period of time [27].

### 2.4. Redox Signaling

Interestingly, nature has already taken advantage of the above-discussed elementary facts, developing during evolution adaptive mechanisms to protect cells under conditions of the increased generation of peroxides. Using careful surveillance systems for the detection of available cytosolic iron levels by the specific sensors IRP1 and IRP2 (iron regulating proteins 1 and 2, respectively) and in cooperation with inflammation and infection signals, cells can finely adjust the existing balance between peroxide tone and labile iron availability [5,28]. When peroxide levels increase, e.g., in the case of inflammation or infection, a fast and robust induction of ferritin eliminates the available iron [10,11] and prevents the formation of damaging HO^•^s or RO^•^s. However, in cases of intense and prolonged oxidative stress, the overall protective capacity of cells can be overwhelmed, thus leading to transduction of a number of different signals, including those of programmed cell death, by either apoptosis or necrosis [10,29].

Apparently, the consequences induced when cells are exposed to peroxides are largely dependent on the type of cells, as well as the level, nature, duration, and location of generated oxidants. Cell responses can range from adaptation to senescence and apoptotic or necrotic death [30,31,32,33,34]. Interestingly, in several cases of oxidative stress-mediated signal transduction (redox signaling), labile iron has been shown to be implicated in the corresponding mechanisms. For instance, we recently showed that labile iron was required for the activation of ASK1-JNK/p38 axis [10,29], which led to apoptotic cell death in Jurkat cells exposed to H_2_O_2_. It is also important to note that H_2_O_2_ freely diffuses through biological membranes and can reach surrounding healthy cells and tissues, imposing oxidative stress on them. On the other hand, the same property enables H_2_O_2_ to act as a signaling molecule in autocrine and paracrine manners.

### 2.5. Labile Iron and Its Pivotal Role in Oxidative Stress-Induced Toxicity

Iron is an essential element for living cells and organisms because it participates in diverse biochemical functions including oxygen transport, cellular respiration, DNA synthesis and repair, and several other enzymatic reactions [28,35]. However, despite its privileged position in living matter, iron participates in damaging free-radical generating reactions known as Fenton-type reactions, in which H_2_O_2_ is converted into the highly reactive HO^•^ via ferryl/perferryl intermediates (Reaction 1).

Reaction 1: Fe^2+^ + H_2_O_2_ → ferryl/perferryl intermediates → Fe^3+^ + HO^•^ + OH^−^

It is obvious that although adequate iron intake is essential for health, iron excess is simultaneously potentially dangerous for cells and tissues [36]. Thus, the tight regulation of iron homeostasis (acquisition, usage, and detoxification) is crucial to avoid both iron deficiency and overload. This need is fulfilled by sophisticated mechanisms that mammals developed to accomplish vital functions and satisfy their metabolic needs for iron while also minimizing its toxicity [37]. Indeed, most of the body’s iron is kept in a redox-inert state. In circulation, iron is tightly bound in the iron carrier transferrin, while most of the intracellular iron is either well-protected in the active sites of enzymes or safely stored in ferritin. However, a small portion of unshielded iron, usually referred to as “labile” or “chelatable” iron, is redox-active, meaning that it can catalyze the generation of HO^•^ via Fenton-type reactions [6,38].

Articulating an exact definition of labile iron is rather difficult. Usually, it is referred to as the fraction of iron that is able to catalyze the generation of HO^•^ and RO^•^ after interaction with peroxides, and, in addition, it can be sequestrated by compounds with a weak chelating capacity [6]. Apparently, the labile iron that is present in biological material can be associated with low affinity binding sites in macromolecules (such as polynucleotides like DNA and RNA, proteins, and lipids) and/or to low molecular weight compounds containing oxygen, nitrogen, and sulfur into their structure [39,40,41].

Thus, the labile iron attached to membrane phospholipids catalyzes the initiation and propagation of lipid peroxidation chain reactions, which can mediate necrotic and ferroptotic types of cell death [5]. On the other hand, iron associated with DNA can induce mutations or single and double strand breaks [42], while iron loosely attached to proteins can promote H_2_O_2_-dependent redox signaling [10,29,43].

Labile iron is not uniformly distributed in various cell compartments, with mitochondria and lysosomes containing higher amounts than to cytosol and the nucleus [44,45]. Consequently, these two organelles are extra sensitive in cases of the increased diffusion of peroxides in their interior. It seems likely that specific, energy-requiring mechanisms are responsible for controlling the correct iron gradients between different cell compartments.

It has to be highlighted here that other transition metals such as copper and nickel can also catalyze the formation of reactive free radicals from the corresponding peroxides even more effectively than iron. However, these metals are found in very low levels and securely chelated in the cells, thus posing no risk or danger [42,46,47], except for in a few special cases of pathological conditions.

## 3. Oxidative Stress and Ageing: The Role of Labile Iron

The rise of human life expectancy in modern societies brought along problems of ageing, associated with the consequent increase in the total burden of morbidity instances. Due to the increasing impact of ageing on the population, intensive research efforts aiming to elucidate the underlining biochemical mechanisms of this process have been made over the last few decades [4]. It is reasonable to expect that actual progress in this direction should open new possibilities for developing novel strategies for the prevention or even treatment of age-related diseases.

### 3.1. The Free Radical Theory of Ageing

The most popular explanation for the molecular basis of ageing is the so called “free radical theory of ageing.” This theory was first proposed in the 1950s by the American gerontologist Denham Harman [2], who stated that “aging and the degenerative diseases associated with it are attributed basically to the deleterious side attacks of free radicals on cell constituents and on the connective tissues.” According to this theory, reactive free radicals arise in vivo as byproducts of enzymatic reactions, catalyzed by trace transition metals such as iron. At that time, the generation of free radicals in vivo was met with skepticism because these species were considered uniformly injurious and incompatible with life. However, the discovery of the actual reaction catalyzed by the SOD enzyme by McCord and Fridovich in 1969 [48], revealed the existence of an intracellular enzyme that uses O_2_^•−^, an oxygen-derived free radical as its substrate, providing convincing evidence for the generation of free radicals in aerobic cells for the first time. This discovery brought the free radical theory of ageing into a new era. Some years later, the focus for the primary site of endogenous oxidant generation was shifted to mitochondria [49], and Harman’s theory expanded to the “mitochondrial free radical theory of ageing” [50].

In support of this theory, evidence that accumulated over the next decades showed that highly reactive oxidants generated by redox reactions have the capacity to non-specifically oxidize all cellular macromolecules, inducing structural modifications that lead to the exposition of hydrophobic surfaces and subsequent aggregate formation [34]. In addition, radical–radical interactions, as well as Schiff base bond formation and Michael additions, contribute to cumulative fixed macromolecular damage over time [51,52].

Indeed, analyses of different samples of human lens and human brain obtained from autopsies/biopsies, human dermal fibroblasts in tissue cultures, and rat liver and whole flies revealed that carbonylated proteins, markers for severe and chronic oxidative stress, were dramatically elevated in the last third of life [53,54]. The oxidative damage of cell constituents is also consistent with other hallmarks of ageing, including the loss of regenerative cell populations due mainly to cell death and senescence, as well as altered cellular communications and genomic instability [55].

Taken together, it is generally accepted that the accumulation of oxidative damage to cellular macromolecules represents a major cause of ageing and age-related chronic diseases. Thus, it is plausible to propose that alterations that are able to modulate the rate of formation of highly reactive oxidants may play decisive roles in modulating the promotion of the ageing process.

### 3.2. Cellular Senescence

Cellular senescence is one of the common markers of organismal ageing. The most prominent characteristic of this fundamental cellular process is the permanent arrest of the cell cycle, which is accompanied by the intracellular accumulation of damaged macromolecules, as well as a secretory phenotype and altered metabolism [55,56]. Two types of cellular senescence have been recognized in mammalian cells; these are referred to as “replicative senescence” and “stress-induced cellular senescence” [56]. The first normally occurs after a certain number of divisions in different types of cells. It was described several decades ago in cultured human fibroblasts [57]. This phenomenon was later attributed to telomere attrition, the gradual shortening of the linear ends of chromosomes upon each DNA replication [58]. On the other hand, stress-induced cellular senescence is largely independent of the telomere length and represents an acute response to numerous stressors including oxidative stress, genotoxic stress, mitochondrial deterioration, hypoxia, nutrient deprivation, and the aberrant activation of oncogenes [56,59,60,61]. Interestingly, oxidative stress is a common denominator for all these cases because it may be involved in all the above-mentioned stressful signals [62,63,64,65].

Cellular senescence is undoubtedly linked with organismal ageing [55,56]. However, senescent cells are not exclusively detected in ageing tissues; they can be detected in any life stage and may play beneficial roles in a broad spectrum of human physiological and pathological processes including embryogenesis, wound healing, and tumor suppression [56,61]. However, the steady accumulation of senescent cells with age has detrimental effects and has been linked to ageing-related diseases and morbidity [56,59,66,67,68,69].

Regarding their morphology, senescent cells show common marks including enlarged, flattened, and irregularly-shaped cell bodies; an altered composition of the plasma membrane; a loss of nuclear condensation; and an increased lysosomal content of senescence-associated beta-galactosidase (SA-β-gal) [70,71]. They also manifest dramatic alterations in their secretory profile, exhibiting an increased expression and secretion of pro-inflammatory cytokines and chemokines, growth factors, components of extracellular matrix (matrix metalloproteinases, serine proteases), and ROS [59]. All these changes are also accompanied by the progressive intracellular accumulation of a biological non-degradable “waste material” that is conventionally called “lipofuscin” or “ceroid” or even “age pigment” [72,73,74].

Subsequent sections describe the mechanistic aspects of lipofuscin formation and propose possible means to hamper or prevent its accumulation.

### 3.3. Lipofuscin Formation and Accumulation in Senescent Cells

The pigment known today as “lipofuscin” was discovered and reported in 1842 by the Dutch histologist Hannover [75]. The term lipofuscin was initially used by Borst in his lectures but was published for the first time by Hueck in 1912 [76,77]. The name was derived from the Greek word lipo (which means fat) and the Latin word fuscus (which means dark or dusky). Lipofuscin formation and accumulation are characteristic changes with universal manifestation in senescent cells [78,79,80] and are more profound in long-lived postmitotic cells, such as neurons, cardiomyocytes, skeletal muscle cells, and retinal pigment epithelial (RPE) cells [74,81]. These cells continue to live normally for a long time after the cessation of their proliferation, but they accumulate gradually increasing amounts of lipofuscin that cannot be degraded or exocytosed.

By using various techniques in order to detect senescent cells, it was observed that the rate of lipofuscin accumulation in similar types of postmitotic cells of different organisms is inversely related to their lifespan [82]. In particular, the rate was rapid in short-lived species and slow in long-lived ones, indicating that lipofuscin accumulation most probably has deleterious effects on cellular functions and is connected to the shortening of an organism’s life span [80,83,84]. Despite the significant importance of this correlation, the exact biochemical mechanisms underlying lipofuscin accumulation, as well as its repercussion on cellular functions, remain poorly understood.

Lipofuscin has been mainly found within lysosomes but also in lesser amounts in the cytosol of aged cells [85,86]. It displays a wide-spectrum of auto-fluorescence with a yellow-brownish color [80,87], but its structure and composition remain poorly defined. Though its composition varies in different cell types, it has been shown to be mainly composed of oxidized proteins and lipids (such as triglycerides, free fatty acids, cholesterol, and lipoproteins) and a small amount of carbohydrates and nucleotide fragments connected to each other by covalent bonds of various types [84]. The attachment of iron on its surface also represents a common characteristic of lipofuscin [88,89].

Though the ultimate effects of lipofuscin accumulation on cellular functions remain unclear, it has been shown that it can inhibit the activities of both proteasomal and lysosomal protein degradation systems. Moreover, there is experimental evidence showing that it can catalyze the further formation of reactive free radicals through redox-active iron ions (labile iron) attached on its surface [89].

### 3.4. Lipofuscin as Over-Oxidized Material in Cells Exposed to Oxidative Stress

**Figure 2:**
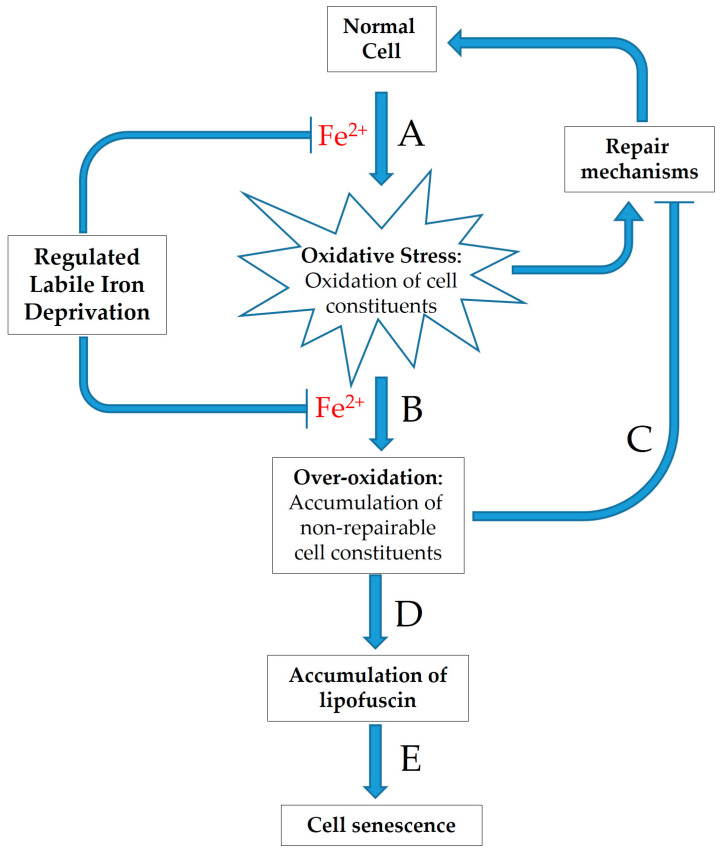
Schematic representation of sequential steps that lead to lipofuscin formation and contribute to cellular ageing. Note that Fe^2+^ is required for the generation of highly reactive ROS (HO^•^ and RO^•^), which are responsible for the oxidation and over-oxidation of cellular macromolecules (**A**,**B**). Over-oxidized macromolecules can inhibit cellular repair systems (especially 20S proteasome), thus facilitating futile cycles of progressively increasing oxidation rates (**C**). Oxidatively modified, non-degradable cellular components are gradually accumulated into cells as covalently interconnected aggregates in the form of lipofuscin (**D**), a fact that is proposed to influence the process of cell ageing (**E**). Arrowheads and flatheads indicate the induction and inhibition, respectively, of processes.

Since lipofuscin comprises a highly oxidized aggregate mainly composed of covalently cross-linked proteins and lipids [90], it is reasonable to postulate that labile iron—able to catalyze the generation of extremely reactive free radicals—is involved in the pathways of its formation [91]. Evidence derived mainly from experimental systems has shown that the exposure of cells to increased levels of oxidative stress invariably leads to the development of a strong senescent-phenotype across different cell-types, with the parallel acceleration of the intracellular formation and accumulation of lipofuscin-like materials [87,89,92,93]. Distinct successive steps leading to lipofuscin formation are illustrated in Figure 2.

As discussed above, the presence of labile iron is required for the generation of highly reactive ROS (HO^•^ and RO^•^), which are responsible for the oxidation and over-oxidation of cellular macromolecules (Figure 2A,B). Moreover, oxidatively modified macromolecules can inhibit protein degradation and cell repair systems, thus facilitating futile cycles of increasing oxidation rates (Figure 2C). The gradual accumulation of over-oxidized, non-degradable cellular components into cells leads to lipofuscin formation (Figure 2D), which is proposed to contribute to cell ageing process (Figure 2E).

Interestingly, Marzabadi et al. [94] observed that lipofuscin accumulation was prevented in iron depleted cells by the use of the iron-chelating drug desferrioxamine, indicating that lipofuscin formation requires highly reactive free radicals such as HO^•^ and RO^•^ (Figure 2). Obviously, these reactive radicals can initiate chain reactions that lead to lipid peroxidation breakdown products, which provoke the formation of the undegradable, non-specific cross-linking of cellular components.

Taken together, the above results indicate that the sensitive equilibrium between the intracellular peroxide level and the available labile iron determines the triggering of a variety of toxic effects that culminate with lipofuscin accumulation, as well as the induction of cellular senescence and cell death by either apoptosis or necrosis [29,95].

The induction of cellular senescence by peroxides can be also achieved through different pathways. For instance, the intermediate rates of H_2_O_2_ cells may directly induce the activation of specific MAP kinases and the transduction of senescence signals, which trigger the activation of the p16INK4aINK4A axis and result in the induction of cell senescence [64,65,92,96]. On the other hand, higher H_2_O_2_ concentrations, as is the case in strongly inflamed areas that attract activated phagocytes, can induce direct iron-catalyzed oxidation on DNA that subsequently trigger senescence signaling pathways. In both cases, the parallel formation and accumulation of oxidatively modified cellular macromolecules represent apparent consequences. It has to be noted, however, that the question of whether lipofuscin accumulation represents a causal factor for cellular senescence or is the consequent of it remains a central but unconcluded question.

### 3.5. Intracellular Iron Homeostasis and Lipofuscin Formation

As discussed above, iron is an essential element for living cells and organisms because it participates in diverse biochemical reactions that support basic functions such as oxygen transport, cellular respiration, and DNA synthesis and repair. However, iron can also be involved in reactions that lead to the generation of damaging free radicals, known as Fenton-type reactions. In order to minimize iron’s toxicity, mammals developed sophisticated mechanisms that regulate its availability [35,37]. Despite that, a small and finely adjusted portion of redox-active iron usually referred to as “labile iron” is always present, presumably representing the actual iron movement between different cell compartments [6,38]. Thus, labile iron represents a dynamic cell parameter that can respond to a variety of stimuli by changing its level, aiming to balance the prevention of cell damage and the guarantee of cell demands.

In conditions of temporarily elevated concentrations of peroxides (conventionally called oxidative stress), labile iron can mediate the following events: (a) the initiation and propagation of lipid peroxidation chain reactions, (b) protein oxidation and cross-linking, (c) the induction of DNA damage such as single and double strand breaks, and (d) the triggering of a variety of complex redox signaling pathways [10,29,43]. All these iron-catalyzed effects can lead to cellular senescence accompanied by the formation and accumulation of lipofuscin.

It is worth stressing here that we have already proven in a series of publications the prevention of H_2_O_2_-induced DNA damage and apoptosis in cells with depleted levels of labile iron by using a variety of iron-chelating agents [11,29,42,43,97]. In these investigations, we used an in vitro cell culture-based experimental system in which different types of human cells were exposed to oxidative stress in the form of H_2_O_2_, and the damage in nuclear DNA was quantitatively estimated by using comet assay, a sensitive method that detects DNA single-strand break formation in individual cells. Interestedly, the pre-incubation of cells with a series of known strong antioxidants such as ascorbic acid, α-tocopherol, Trolox, N-acetylcysteine, and α-lipoic acid before the exposure to H_2_O_2_ did not offer any protection [7]. Since the capacity of these agents to combat free radicals has been established in numerous in vitro studies, the above-mentioned negative results were attributed to the inability of these agents to effectively scavenge the reactive free radicals generated inside the cells.

An important parameter of iron-catalyzed cross-linking may be the facilitation of the covalent binding of oxidized soluble cell components to biological membranes. Such an event should hamper the exocytosis of the membrane attached materials, leading to its permanent intracellular accumulation. It is reasonable to speculate that lysosomal membranes should be primary targets in this case due to their proximity with the location of lipofuscin formation. Indeed, lipofuscin has often been detected inside cells embraced by lysosomal membrane segments [98].

Given the importance of available labile iron for the formation and accumulation of lipofuscin, the regulation of its intracellular homeostasis seems to be of utmost importance regarding the ageing process. The appreciation of labile iron availability as a pivotal factor that determines the oxidation and over-oxidation of cell components and the accumulation of lipofuscin in cells may open the road for the development of novel strategies, aiming to interfere and modulate the biological clock of the ageing process.

### 3.6. Inactivation of Repair Systems by Over-Oxidized Cell Components

Cell strategies for the repair of different oxidized cell components vary widely, depending on the nature of the particular components. For instance, oxidized DNA nucleotides are removed and replaced by normal ones through a process called “nucleotide excision repair,” while oxidized proteins are degraded to single amino acids that can then be reused for new protein synthesis.

There are several different protein degradation systems: in cells, there are lysosomal enzymes; in cytosol, there are proteasomes and calpains; in the mitochondrial matrix, there are the Lon proteases (ATP-dependent proteases); and in the mitochondrial membrane, there are the triple A proteases [78,98,99,100]. Moreover, in addition to oxidatively modified proteins, lysosomes can also take up and degrade even heavily damaged organelles such as mitochondria or part of the cytoplasm in processes called chaperon-mediated autophagy, macro-autophagy, and micro-autophagy [82,101].

Despite the fact that most oxidatively modified biomolecules and organelles can be efficiently repaired or degraded by cells, it has been observed that some of them accumulate with age, suggesting the inherent inadequacy of the cellular turnover mechanisms. It has been shown that already oxidized cell components can undergo further oxidative modifications, leading to the formation of products that cell degradation systems are incapable to cope with [34,84]. The accumulation of such non-degradable conglomerates can, in turn, hamper the functionality of degradation systems, thus aggravating effects and leading to a vicious cycle, as schematically illustrated in Figure 2.

In cases of increased and long-lasting oxidative stress conditions, the repair capacity of cells in general and the protein degradation capacity in particular can reach saturation levels, thus leading to the persistent presence of oxidized components. This situation increases the probability of the further oxidation of already oxidized components and the formation of additional and more profound oxidative modifications, including intra- and intermolecular covalent bond formations. The overall complexity of the formed chemical structures exceeds the degradation ability of cellular proteolytic systems (especially the 20S proteasome), leading to the gradual accumulation of over-oxidized undegradable “garbage” materials inside the cells, mainly into lysosomes [82,102].

Taking together, the accumulation of over-oxidized materials inside cells increases the probability of the further oxidation of already oxidized cell components over time, thus facilitating the initiation of a vicious cycle of oxidation, over-oxidation, and accumulation; all of these ultimately lead to the progressive impairment of cellular functions, as is apparent in ageing and senescence.

### 3.7. Lysosomes as the Main Sites of Lipofuscin Formation

As an outcome of normal autophagic degradation, the lysosomal compartment is extra rich in labile iron since many auto-phagocytized macromolecules and organelles contain iron. The combined presence of redox-active iron and low pH in lysosomes facilitate the formation of extremely reactive radicals from relatively unreactive peroxides via the Fenton reaction. Therefore, this organelle is extra sensitive to the mild oxidative stress that cells naturally experience during the transient fluctuation of the intracellular H_2_O_2_ steady state. The generated HO^•^s instantly induce chain oxidations of lysosomal components, such as proteins and membrane lipids, leading to the formation of lipofuscin-like materials that indeed have been shown to be accumulated in lysosomes.

In cases of intense and long-lasting oxidative stress conditions, the simultaneous presence of H_2_O_2_ and labile iron induces further oxidation on top of already oxidized au-to-phagocytosed biomolecules, leading to over-oxidized products that are cross-linked with multiple covalent bonds. This material, in addition to being resistant to degradation, can inhibit cell reparation systems, as has been proven in proteasomes [85,102]. This proposal is strongly supported by the observation that the combination of oxidative stress with the inhibition of lysosomal proteases delayed the degradation of auto-phagocytosed macromolecules and provided more time for their oxidation, dramatically accelerating lipofuscin formation in cultured cells [7].

Lipofuscin itself can originate from different types of auto- or hetero-phagocytosed material. In many cells, especially in highly aerobic ones such as cardiac myocytes and neurons, auto-phagocytosed mitochondria constitute the bulk of intra-lysosomal undegradable material. Strong evidence for the mitochondrial origin of significant part of lipofuscin body represents the observation that abundant ATP synthase subunits are present in lipofuscin-loaded cells [103]. However, in professional scavenger cells with active phagocytosis such as macrophages, microglial cells, and retinal pigment epithelial cells, a substantial portion of their lipofuscin contents may also be derived.

### 3.8. Detection of Senescent Cells

The recognition of senescent cells is a critical issue given the increasing evidence of the role of senescence in human pathologies [56,104]. Furthermore, the rapidly expanding field of senotherapeutics requires the precise detection of senescent cells [105]. Various markers detecting sensors of cellular senescence are presented in Table 1. Recent findings have indicated the implication of senescence in COVID-19, justifying the application of senotherapeutics for the treatment or prevention of COVID-19 patients [106].

**Figure 3:**
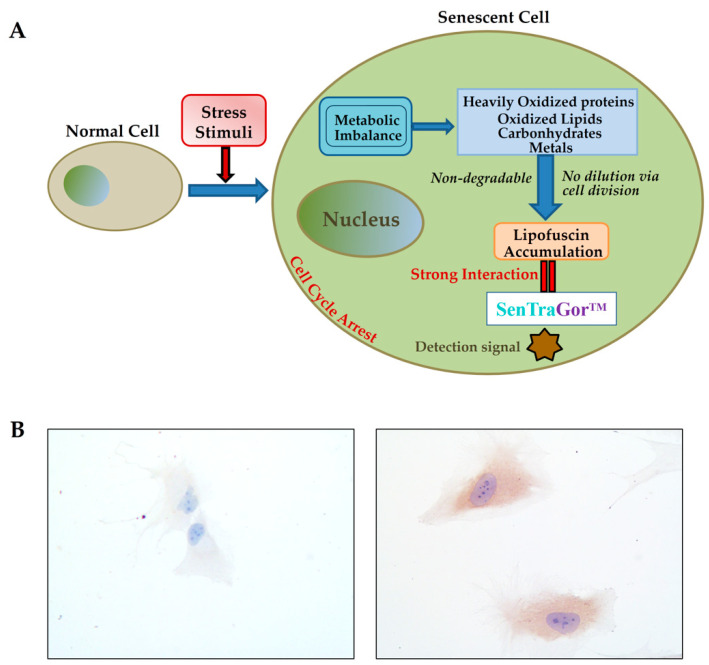
(**A**) SenTraGorTM specifically reacts against lipofuscin, the non-degradable byproduct of cellular senescence, allowing for the accurate identification of senescent cells in vitro and ex vivo by applying an antibody-mediated detection method. (**B**) SenTraGor staining on Li-Fraumeni-p21WAF1/Cip1 Tet-OFF (left image) and OΝ cells (right image); original magnification: ×200.

The accumulation of newly formed lipofuscin can be detected and quantified by using electron, confocal, and fluorescence microscopy, as well as flow cytometry [108,109]. Moreover, lipofuscin can be detected on the base of its autofluorescence in combination with a number of histochemical and cytochemical techniques [68,87,110,111]. Particularly, GL13, a biotinylated Sudan Black-B (SBB) chemical analog that is commercially available as “SenTraGorTM,” interacts with lipofuscin and allows for the accurate identification of senescent cells in vitro and ex vivo by applying an antibody-mediated detection method [56,107,110]. Employing this assay, the quantitative determination of soluble or extracted lipofuscin levels in cell culture supernatants, body fluids, and tissue homogenates is also achievable [112]. The sequence of events leading to lipofuscin accumulation during senescence and its interaction with lipofuscin is schematically presented in Figure 3A. Representative images of Li-Fraumeni-p21WAF1/Cip1 Tet-OFF and ON (senescent) cells, stained with SenTraGor, are presented in Figure 3B. A strong brown cytoplasmic signal is evident in senescent cells (right image), while no induced cells are negative (left image).

The development of theranostic applications based on nanotechnology might allow for the accurate targeting of senescent cells [113,114,115]. The mapping of the senescent cells in vivo remains a great challenge. In this context, the novel GL13 compound might be enriched by the incorporation of quantum dots or other appropriate nano-carriers and a hydrophilic hull to encapsulate the whole system, rendering GL13 a promising candidate for molecular imaging in vivo [114].

## 4. Dietary Bioactive Compounds and Oxidative Stress

Numerous epidemiological studies conducted mainly during the second half of the previous century have correlated the traditional Mediterranean diet (the diet that prevailed in the north shores of the Mediterranean basin) with lower incidences of certain chronic diseases and reduced morbidity and mortality risks [116,117,118]. Hence, intense research efforts have been carried out to identify Mediterranean diet agents that are able to prevent or attenuate the deleterious effects of oxidative stress and to delineate their molecular mode of action.

### 4.1. Dietary Bioactive Compounds: Free Radical Scavenging Antioxidants or Weak Iron Chelators?

The traditional Mediterranean diet is characterized by a high consumption of olive oil and plant foods such as fruits, vegetables, unrefined cereals, and legumes; a moderate consumption of fish, dairy products, and wine; and a low consumption of meat products [119]. Its health benefits have been frequently attributed to the high quantities of antioxidants of the free radical scavenger type, which are largely present in typical foods of this diet. It was generally assumed that such free radical scavengers can interact with and neutralize free radicals, thus combating oxidation in the body and consequently delay or even prevent the incidence of various chronic diseases, including the ageing process [120,121,122,123].

However, the results of the largest clinical trials of antioxidant supplementation conducted thus far have failed to show substantial protection against the development of chronic diseases [124,125,126,127,128,129,130,131,132,133,134,135,136,137]. Furthermore, concerns have been raised about the safety of the high-dose supplementation of antioxidants because links with health risk were observed in some instances [138,139].

**Figure 4:**
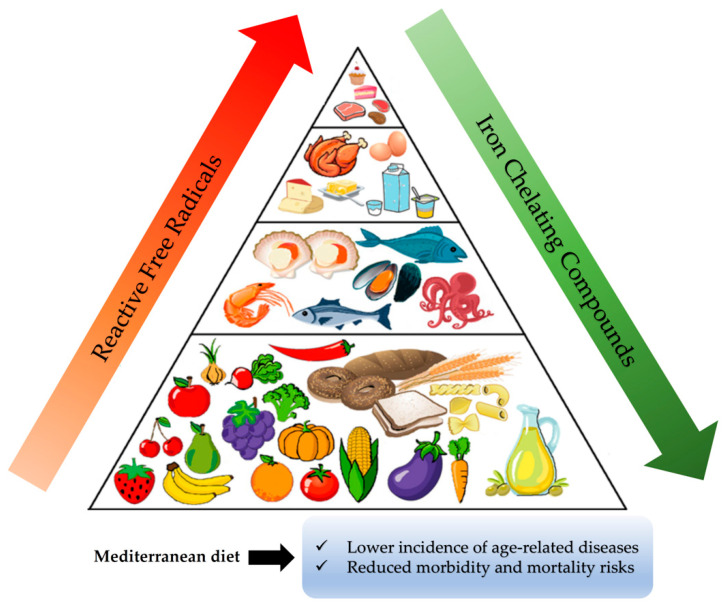
Schematic presentation indicating that the plant-derived foods of the Mediterranean diet contain increasing amounts of iron-binding compounds able to chelate intracellular labile iron and to prevent the generation of highly reactive free radicals that are responsible for the unregulated oxidation of cell constituents.

This failure can be explained by the fact that free radicals such as HO^•^ and RO^•^ are extremely reactive, instantly and non-specifically attacking and oxidizing every chemical group present in the vicinity of their generation [140]. Thus, when generated inside cells, it is practically impossible for any externally derived free radical scavenger to neutralize them. It has to be emphasized here that the only chance to protect cell constituents from oxidation and damage under oxidative stress conditions is to prevent the generation of such highly reactive free radicals. Another possible strategy to avoid the oxidation of critical biological macromolecules like DNA and proteins under such circumstances could be to manipulate the location of their formation by using iron-chelating agents. As is discussed below, diet in general and the Mediterranean diet in particular contain a plethora of such weak iron chelators, (Figure 4) that, when able to pass through the cell membrane, can detach weakly bound labile iron from important macromolecules, thus protecting them from undesirable oxidation regardless of whether they inhibit the Fenton reaction or not.

Typical foods of the Mediterranean diet contain numerous compounds, including phenolic alcohols, phenolic acids, and flavonoids, which have been repeatedly proposed to act as free radical scavenging antioxidants. A number of such compounds have been examined by our research group, and we observed a strong relationship between the protective capacity of each compound and its ability to chelate intracellular labile iron but not with their ability to scavenge free radicals in vitro [8,9,12]. An additional necessary property of these compounds that was required to exert their protection capacity, was their ability to reach cell interior by diffusion or any other kind of transport through the plasma membrane [11,42,141].

Based on these observations, we proposed that bioactive compounds ubiquitously present in the Mediterranean diet offer their cytoprotective effects by detaching intracellular labile iron from critical cellular constituents, thus diminishing their undesirable oxidation.

### 4.2. Does Dietary Iron-Chelating Agents Prevent Lipofuscin Formation?

Based on the above-mentioned considerations, it is reasonable to speculate that bioactive iron-chelating agents present in the Mediterranean diet may represent key factors that are responsible for the prevention of lipofuscin formation and, consequently, the ageing process in general. As far as we know, systematic efforts aiming to experimentally test this important hypothesis have not been performed yet.

A great number of iron-chelating molecules with different chemical structures and characteristics are contained in a typical Mediterranean diet. For instance, we have extensively studied plant extracts containing numerous polyphenols and have established that phenolic compounds with an ortho-dihydroxyl group are protective against oxidative stress, while those lacking one hydroxyl or having it located in a meta- or para-position are entirely ineffective [8,10,11,12]. These observations raised the additional question of whether the iron-chelating agents contained in foods are capable of penetrating several barriers in order to reach the interior of the target cells. In this case, the particular dietary agents can be considered as “indirect antioxidants” because they prevent the generation of reactive free radicals rather than detoxifying them after their intracellular production.

In some instances, intracellular labile iron ions can be incompletely coordinated with diet-derived agents due to their low uptake and considerable dilution in the body, thus permitting the engagement of iron in redox reactions. Nevertheless, the same agents usually possess dual functions because they can comprise both iron-binding and free radical-scavenging properties in the same molecule. Hence, diet-derived iron chelators may function in a dual manner: either mitigating oxidative stress-induced cell damage by removing loosely bound labile iron from vulnerable cellular macromolecules and fully inactivating it or by the incomplete coordination of iron, which results in its removal from its original position but allows it to remain redox-active and able to oxidize the corresponding diet-derived iron chelators.

## 5. Conclusions

One of the most prominent concepts in the area of ageing today is the so called “free radical theory of ageing.” According to this theory, organismal ageing is caused by cumulative oxidative damage inflicted by highly reactive free radicals that primarily arise as a consequence of aerobic metabolism. The continuous generation of such extremely reactive radicals causes the gradual formation and accumulation of non-repairable aggregates of damaged cell constituents. This chemically undefined material, which mainly consists of proteins and lipids and which exerts a yellow-brown fluorescence, is known as “lipofuscin,” “ceroid,” or “age pigment,” and it is considered a hallmark of cellular ageing.

Lipofuscin is mainly formed through uncontrolled and nonspecific oxidative modifications of cellular macromolecules. Cells are equipped with multifaceted defense systems to surveil and repair oxidized macromolecules. However, when intense oxidative stress persists for extended periods of time, it invariably results in the generation of highly reactive free radicals and in the over-oxidation of already oxidized materials, thus creating products that are unable to be repaired, degraded, or even exocytosed by the relevant cellular systems. Moreover, it has been shown that over-oxidized materials can induce a gradual inactivation of cellular protection and reparation systems, thus fueling futile cycles of increased rates of lipofuscin accumulation.

Since highly reactive free radicals can be generated in iron-catalyzed oxidation processes (Fenton reaction), the availability of labile iron represents a necessary precondition for lipofuscin formation and accumulation inside the cells. Based on these considerations, it is plausible to speculate that the fine regulation of cellular iron homeostasis in general and labile iron distribution in particular may represent a hitherto unappreciated manner to retard intracellular lipofuscin formation and consequent cellular ageing (senescence).

We have previously shown that a number of iron-chelating phytonutrients contained in the Mediterranean-type of diet are able to penetrate biological membranes and reach cell interiors [8,9,11,12]. These agents chelate intracellular labile iron (not necessarily with high affinity) and thus determine its distribution and, consequently, the locations of oxidative stress-induced oxidation. According to the proposed mechanism, diet-derived phytochemicals must combine the following characteristics in their structure in order to be able to protect cells in conditions of oxidative stress: they must be able (a) to penetrate cellular membranes; (b) to chelate cellular labile iron; and (c) in the case of an interaction of the bound iron with peroxides (the incomplete occupation of its coordination sites), to scavenge the formed reactive radical.

Summarizing the conclusions from the above presentation, the following statements can be made: (a) labile iron represents the main agent that is responsible for the production of highly reactive free radicals that are able to oxidize cellular constituents under conditions of oxidative stress, (b) oxidized and especially over-oxidized cell components comprise the main body of lipofuscin that is formed and accumulated inside the cells under these conditions, (c) the depletion of intracellular labile iron by iron-chelating agents prevents the oxidation of cellular components, and (d) our diet and especially the Mediterranean-type diet contain plethora of compounds that are able to modulate intracellular iron distribution.

Considering the above considerations together, it is reasonable to expect that the identification of bioactive nutritional compounds with the assigned properties may allow for their use as pharmacological tools for concrete protective actions in conditions of increased oxidative stress in cells, tissues, and whole organisms. This proposal might open new roads for development of strategies aiming to slow down the rates of appearance and development of age-related diseases.

## Figures and Tables

**Table 1:** Detection markers of cellular senescence sensors [107]. Up arrows indicate an increase, down arrows, a decrease.

Sensors	Detection Markers
Lipofuscin	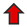 GL13, Sudan Black-B (SBB)
Senescence-associated beta-galactosidase (SA-β-gal)	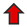 Beta-galactosidase
Tumor suppressors and cell cycle regulators	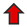 p16, p21, p53
DNA damage response (DDR) sensors	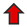 p-ATM, p-53BP1, p-γH2AX, p-NBS1, p-CHK2
Proliferation Sensors	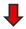 Ki-67, BrdU/EdU-incorporation
Senescence-associated secretory phenotype (SASP) sensors	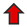 Interleukin 6 (IL-6), interleukin 8 (IL-8), CCL2, MMPs
Other sensors	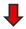 Lamin B and 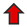 HMGA
